# Epigenetic regulation espeically histone modifications in breast cancer: A viable and emerging targeted therapeutic strategy

**DOI:** 10.7150/jca.119306

**Published:** 2025-09-22

**Authors:** Yibing Zhou, Haotian Liu, Weimin Hong, Haotian Su, Yuxiao Mu, Yijie Cheng, Chaoshen Wu, Xuli Meng, Da Qian

**Affiliations:** 1 Central Laboratory, Department of Scientific Research, Changshu Hospital Affiliated to Soochow University, Changshu No.1 People's Hospital, Changshu, 215500, Jiangsu Province, China.; 2 General Surgery, Cancer Center, Department of Breast Surgery, Zhejiang Provincial People's Hospital (Affiliated People's Hospital), Hangzhou Medical College, Hangzhou, 310014, Zhejiang Province, China.; 3 Department of Pharmacy, The Third Affiliated Hospital (The Affiliated Luohu Hospital) of Shenzhen University, Shenzhen, 518001, China.; 4 Department of Pharmacy, Changshu Hospital Affiliated to Soochow University, Changshu No. 1 People's Hospital, Changshu, 215500, Jiangsu Province, China.

**Keywords:** breast cancer, epigenetic regulation, histone modification, histone target inhibitors, DNA methylation inhibitors

## Abstract

Epigenetic regulation, encompassing DNA methylation, histone modifications, and non-coding RNA activities, is a crucial mechanism through which gene expression is modified without corresponding changes in genomic DNA sequences. Dysregulation of these mechanisms can lead to histone and DNA modifications that either suppress or enhance the expression of disease progression-related genes. Among these regulatory processes, histone modifications are particularly significant, as they contribute to genomic stability, DNA repair, and chromatin dynamics, all of which influence breast cancer initiation and progression. This review offers a detailed analysis of the current state of research centered on epigenetic regulatory factors, with a particular focus on the role that histone modifications play in the treatment of breast cancer. It also examines the interplay between epigenetic modifications and the effectiveness of radiotherapy and chemotherapy when treating breast cancer. Lastly, this article explores the potential of epigenetic regulatory factors as viable targets for the future design of new anticancer therapies.

## 1. Introduction

Breast cancer is among the most widely diagnosed malignancies and remains a prominent driver of global cancer-related mortality in women 1. GLOBOCAN 2022 indicates that 2.31 million new breast cancer diagnoses throughout the world in 2022, comprising 11.6% of all new cancer diagnoses. Over this same period, breast cancer was responsible for 666,000 deaths, comprising 6.9% of all cancer-related fatalities worldwide 2, 3. Breast cancer subtypes are defined by the expression of the major biomarkers estrogen receptor (ER), progesterone receptor, human epidermal growth factor receptor 2 (HER2), and Ki67 4. The multidisciplinary treatment approach for operable breast cancer integrates local interventions, including surgery and radiotherapy, with systemic therapies, which include hormonal treatments, chemotherapy, and molecularly targeted agents. These systemic therapies can be administered as monotherapies or in combination regimens to enhance efficacy 5. Despite advances in treatment, breast cancer remains a complex disease, often characterized by drug resistance and severe adverse effects associated with conventional therapies, which contribute to suboptimal patient outcomes 6. The integration of epigenetic-targeting agents, such as inhibitors of histone modification, with existing therapeutic modalities offers a promising strategy for improving treatment efficacy and overcoming drug resistance 7.

## 2. Epigenetic Regulatory Mechanisms

**Figure 1:**
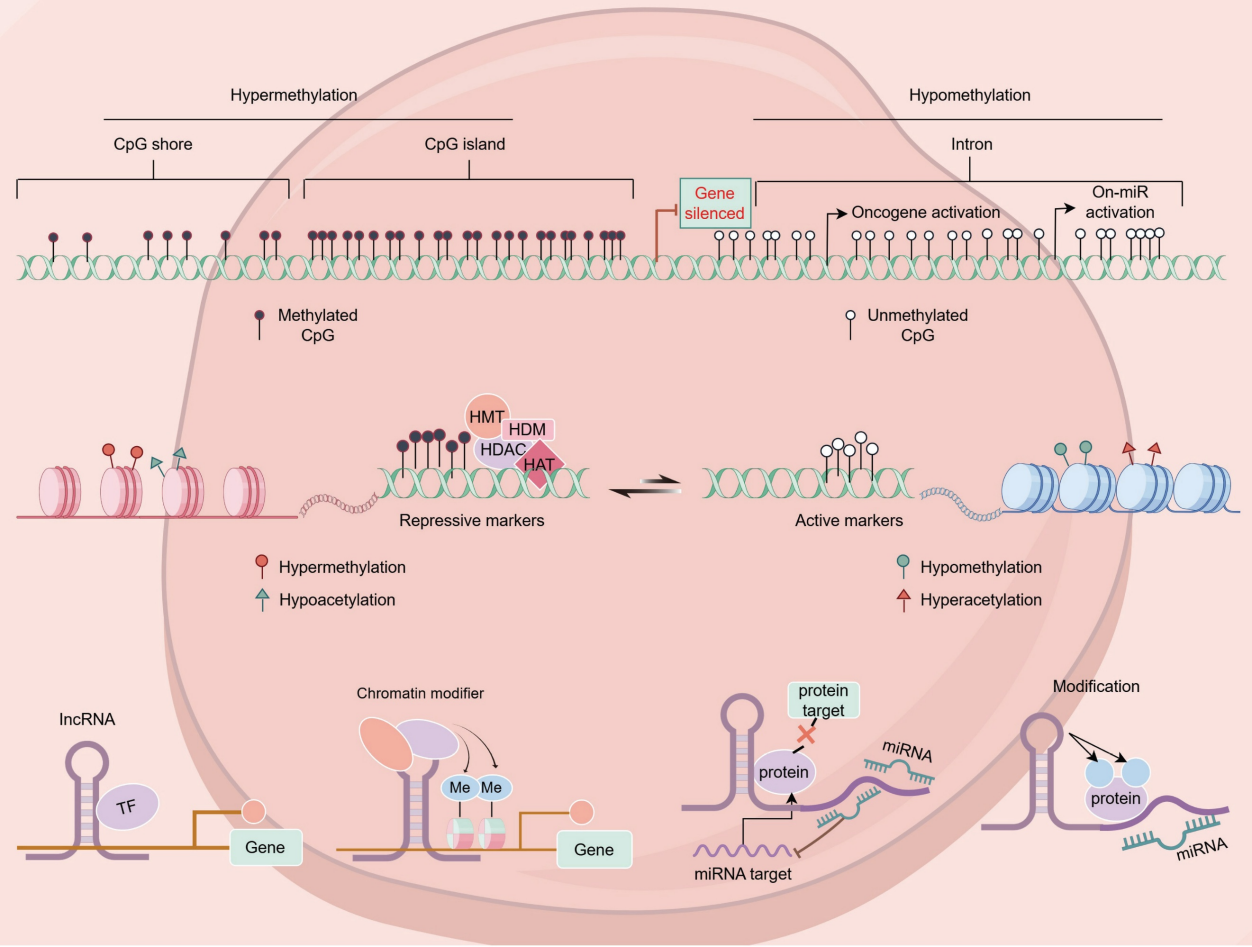
Various epigenetic alterations in breast cancer.

Epigenetics consists of a class of heritable, reversible modifications capable of regulating gene expression without affecting the sequence of the underlying DNA. These mechanisms can entail DNA methylation, histone modifications, non-coding RNA activities, the remodeling of the chromatin, and nucleosome positioning 8. In breast cancer, abnormal epigenetic modifications can result in tumor suppressor gene silencing and oncogene activation, thereby promoting tumor onset and progression 9 (Figure 1).

### 2.1 Breast cancer-related histone modifications

Histone modifications entail covalent chemical changes at the N-terminal histone tails, and can consist of phosphorylation, methylation, acetylation, and ubiquitination, among other post-translational modifications 10. The resultant effects modulate chromatin structure and transcriptional activity, ultimately affecting gene expression. Dysregulated histone modifications contribute to aberrant gene expression in breast cancer, thereby facilitating tumor progression 11.

#### 2.1.1 Histone acetylation

Histone acetylation is a process through which acetyl groups (-COCH₃) are enzymatically added to lysine residues, primarily on histones H3 and H4. Key acetylation sites can include H3K9, H3K14, H3K27, H4K5, H4K8, H4K12, and H4K16 12. This process is dynamically regulated by histone acetyltransferases (HATs), which promote acetylation, and histone deacetylases (HDACs), which remove acetyl groups 13. Acetylation relaxes chromatin structure, enabling RNA polymerase and transcription factors to bind the DNA, leading to enhanced gene transcription, whereas deacetylation is related to gene silencing 14.

In breast cancer, histone acetylation is a major driver of tumor development through its ability to modulate gene expression. A notable observation in cancer is the global reduction of H4K16 acetylation, which is thought to occur early in breast tumorigenesis 15. Suzuki et al. reported significantly lower ac-H4 and ac-H4K12 levels in ductal carcinoma in situ and invasive ductal carcinoma as compared to the normal mammary epithelium 16. Additionally, H3K4ac has been linked to both early- and late-stage breast cancer, as it is highly enriched at gene promoters involved in cancer-related pathways, including estrogen response and epithelial-mesenchymal transition 17. Beyond regulating oncogenes or tumor suppressor genes, abnormal histone acetylation is implicated in other biological processes relevant to breast cancer, such as DNA repair, metastasis, apoptotic death, metabolic activity, cellular homeostasis, cell cycle regulation, cell adhesion, and cellular proliferation 18.

#### 2.1.2 Histone methylation

Histone methylation occurs on arginine, lysine, and histidine residue side chains, influencing gene expression through distinct methylation patterns. Lysine residues can undergo mono-, di-, or tri-methylation, while the asymmetric or symmetric methylation of arginine residues can take place. The types of histone methylation most commonly reported in the literature are H3K4, H3K9, H3K27, H3K36, H3K79, and H4K20 19. Different methylation states are associated with distinct genomic regions. For example, H3K4me2/3 is typically found at transcription start sites (TSS) of actively transcribed genes, while H3K4me1 is associated with active enhancer regions 20. Consistently, H3K9me1 is related to active gene transcription, whereas H3K9me3 is linked to gene repression 21.

The tumor suppressor gene p53, which is frequently mutated or dysregulated in breast cancer, plays a crucial role in preventing oncogenesis 22. H3K4 trimethylation at the p53 promoter region is related to its enhanced expression 23, while H3K27 trimethylation may play a role in its silencing 24. These histone modifications can impair p53 function, promoting breast cancer progression. BRCA1, among the most well-established breast cancer susceptibility genes 25, is also regulated through histone methylation. H3K27me3-mediated methylation of the BRCA1 promoter is linked to its downregulation, increasing susceptibility to breast cancer development 26, 27.

#### 2.1.3 Histone phosphorylation and ubiquitination

Beyond acetylation and methylation, histone phosphorylation and ubiquitination also play vital roles in the control of chromatin dynamics and the expression of genes. Histone H1 phosphorylation is closely linked with chromatin relaxation during interphase and its condensation in mitosis 28.

Histone ubiquitination, primarily facilitated by the Polycomb Repressive Complex 1 (PRC1), is integral to gene silencing in human cells 29. PRT4165 is a potent inhibitor of PRC1-driven H2A ubiquitination, both in vivo and in vitro 30. Functional assays of E3 ubiquitin ligase activity indicate that PRT4165 directly inhibits RNF2 and RING1A, key components responsible for the enzymatic activity within the PRC1 complex. Given its ability to suppress PRC1 function, PRT4165 presents a promising target for anticancer therapy 31.

### 2.2 Breast cancer-associated DNA methylation

DNA methylation is among the most widespread epigenetic modifications, typically occurring within CpG islands 32. In normal cells, methylation patterns remain relatively stable; however, in breast cancer, genome-wide hypomethylation coupled with hypermethylation at specific promoter regions is frequently noted 33. The aberrant hypermethylation of CpG islands in tumor suppressor gene promoters leads to transcriptional silencing, contributing to breast cancer initiation and progression 34.

In the context of breast cancer, multiple genes undergo significant methylation alterations 35. Key tumor suppressor genes, including BRCA1, p16, and RASSF1A, often exhibit hypermethylation, leading to their silencing and subsequent loss of tumor-suppressive functions. In contrast, oncogenes such as c-Myc and cyclin D2 frequently undergo hypomethylation, leading to their overexpression and promoting uncontrolled cell proliferation and survival. Furthermore, the hypermethylation of DNA repair genes like MGMT and MLH1 exacerbates genomic instability 9.

DNA methylation markers provide several advantages over other types of tumor biomarkers in the context of breast cancer diagnostics, including improved sensitivity, non-invasiveness, and early detection 36. Notably, aberrant methylation patterns can be identified in early-stage breast cancer and even in precancerous lesions 37. For example, hypermethylation of APC and RASSF1A has been detected in breast cancer patient blood samples, highlighting their potential as non-invasive diagnostic markers 38, 39. The integration of DNA methylation profiling with other omics data has led to significant improvements in early cancer screening accuracy 40.

The methylation status of specific genes serves as a valuable prognostic indicator for breast cancer patients. For instance, BRCA1 promoter hypermethylation is related to poor triple-negative breast cancer patient outcomes, while ESR1 gene methylation may predict responsiveness to endocrine therapy 41, 42. These epigenetic markers hold great potential for guiding personalized treatment strategies.

### 2.3 miRNA expression in breast cancer

MicroRNAs (miRNAs) are small, non-coding RNAs, typically 21-23 nucleotides in length, that play vital roles in post-transcriptional gene regulation 43. Some miRNAs are located within fragile genomic regions and are frequently subject to deletions or amplifications, resulting in their abnormal expression in various cancers, including breast cancer 44. Depending on their function, miRNAs can function as tumor suppressors or oncogenic miRNAs (oncomiRs), influencing key pathways involved in cancer progression 45.

The first established link between miRNAs and cancer was demonstrated by Croce et al., who found that miR-15 and miR-16-1 can suppress tumor development via targeting the anti-apoptotic protein Bcl-2 46. In breast cancer, more than 40 miRNAs have been identified as key regulators, with the dysregulation of these miRNAs and their targets exhibiting either tumor-promoting or tumor-suppressing properties 47. Notably, miR-126 and miR-335 suppress metastasis in breast cancer models in vivo 48.

miRNAs also hold promise as biomarkers for breast cancer subtyping, metastasis prediction, and therapy resistance assessment 49. While chemotherapy remains a cornerstone of metastatic breast cancer treatment, a significant subset of patients fails to respond to standard chemotherapy or endocrine therapies 50. Studies indicate that bone metastases have been associated with the presence of miR-10a and miR-10b in breast cancer 51. Similarly, plasma levels of miR-210 that are elevated are related to trastuzumab sensitivity, tumor burden, and lymph node metastasis 52. Identifying circulating miRNAs capable of early cancer detection or predicting therapeutic response could revolutionize breast cancer treatment, ultimately improving patient outcomes.

### 2.4 Chromatin remodeling and nucleosome positioning

Chromatin remodeling is driven by ATP-dependent chromatin remodeling complexes that modulate nucleosome positioning to regulate gene transcription. Nucleosomes, which serve as the fundamental chromatin units, are composed of DNA that wrap around histone octamers, facilitating DNA organization and protection within the nucleus. The positioning of nucleosomes determines the precise genomic arrangement of nucleosomes throughout chromatin landscapes 53. The four major chromatin remodeling complex families in humans are SWI/SNF, ISWI, CHD, and INO80 54. Among these, BRG1, a core subunit of the SWI/SNF complex, has been implicated in breast cancer progression. Research has demonstrated the overexpression of BRG1 in invasive ductal carcinoma, with high BRG1 levels correlating with reduced overall survival and recurrence-free survival in breast cancer patients 55. Despite these findings, research on the role of chromatin remodeling complexes in breast cancer remains limited, underscoring the need for further in-depth investigations.

## 3. Epigenetic Drugs in the Treatment of Breast Cancer

### 3.1 Histone target inhibitors

**Figure 2:**
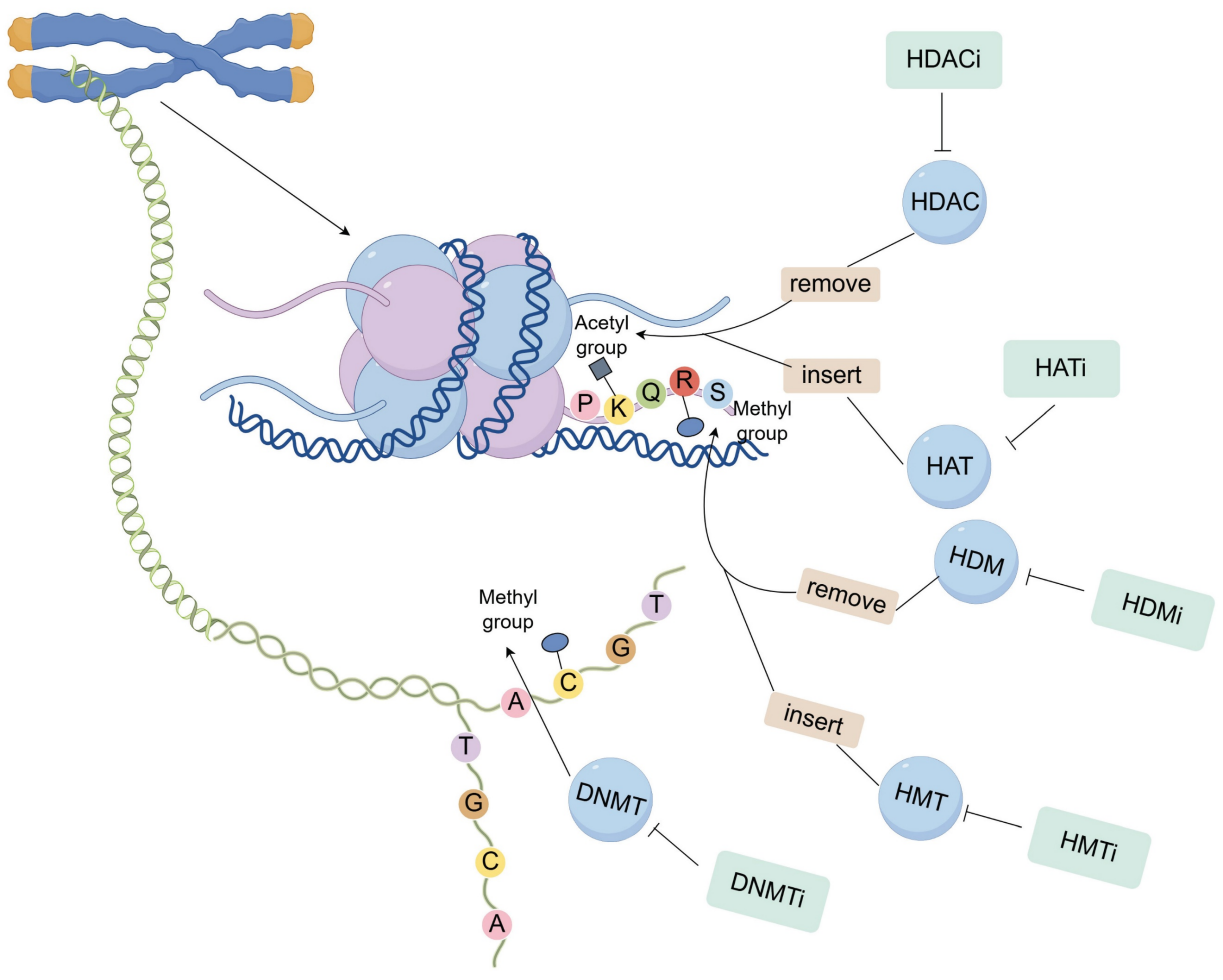
Histone targeting inhibitors agents exert their anti-neoplastic effects in breast cancer by specifically inhibiting various classes of histone-modifying enzymes.

Histone targeting inhibitors represent a class of pharmacologically active compounds that modulate gene expression, chromatin architecture, and cellular differentiation through the regulation of histone function and post-translational modifications. These therapeutic agents exert their anti-neoplastic effects in breast cancer by specifically inhibiting various classes of histone-modifying enzymes (Figure 2).

#### 3.1.1 Histone deacetylase inhibitors (HDACi)

HDACs are often overexpressed in tumor cells, leading to excessive histone deacetylation, which in turn suppresses the transcription of essential housekeeping and tumor suppressor genes. This epigenetic silencing plays a role in malignant transformation 56. HDAC inhibitors (HDACi) counteract this process by restoring tumor suppressor gene expression, resulting in the inhibition of tumor cell proliferation as well as the induction of apoptosis 57. With over 50 HDAC inhibitors developed, they represent the largest class of epigenetic drugs and account for most FDA-approved epigenetic therapies for cancer 58. Clinically, HDACi have demonstrated efficacy against various malignancies, including pancreatic, ovarian, breast, colon, prostate, and thyroid cancers 59-64.

HDACi are classified based on their chemical structure into short-chain fatty acids, benzamides, cyclic peptides, and hydroxamic acids 65. A well-known example of a short-chain fatty acid inhibitor is butyrate, which, at higher doses, can suppress growth in multiple cancers, including colorectal, prostate, breast, endometrial, and cervical cancers 66. Sodium butyrate has been found to exert both anti-proliferative and pro-apoptotic effects in breast cancer cells while modulating genotoxicity 67. Entinostat, a synthetic benzamide-derived HDACi, enhances the expression of ER and aromatase, thereby restoring sensitivity to nonsteroidal aromatase inhibitors (NSAIs). A Phase 1 clinical trial found that the combination of entinostat with exemestane improved tolerability and antitumor efficacy in hormone receptor-positive (HR+) advanced breast cancer (ABC). The Phase 2 ENCORE301 trial, which included HR+ ABC patients who had progressed following endocrine therapy but had not received CDK4/6 inhibitors, revealed significantly improved progression-free and overall survival (PFS and OS) with the entinostat-exemestane combination compared to exemestane alone. Moreover, a multicenter Phase 3 clinical trial conducted in China demonstrated that entinostat significantly prolonged PFS in HR+/HER2- ABC patients following endocrine therapy failure 68. Cyclic peptide HDAC inhibitors, such as Romidepsin (FK228, depsipeptide), have also been investigated in breast cancer. Romidepsin has completed a Phase 2 clinical trial for metastatic breast cancer (NCT02393794). In patients treated with Romidepsin combined with Cisplatin and the PD-1 inhibitor Nivolumab, the median progression-free survival (PFS) was 4.4 months, with a 1-year PFS rate of 23%. The median overall survival (OS) was 10.3 months, and the 1-year OS rate was 43%, showing significant efficacy 69, 70. Studies indicate that FK228 and its analogs act as dual inhibitors of HDAC and PI3K, directly inhibiting PI3K activity while promoting apoptosis 71. Hydroxamic acid-based HDACi, such as Vorinostat (SAHA) and Panobinostat, have been explored as ER+ breast cancer therapies. Vorinostat has demonstrated efficacy in enhancing anti-estrogen therapy and has shown promising outcomes in Phase 2 trials (NCT00365599) in patients resistant to tamoxifen or aromatase inhibitors. The efficacy is particularly more significant in patients with high histone acetylation or high HDAC2 expression. However, hematological toxicity requires close monitoring, and future efforts should focus on exploring biomarker-guided precision treatment strategies 72. Panobinostat has been found to downregulate BRCA1 expression in breast cancer, thus sensitizing tumor cells to PARP inhibitors (PARPi) 73.

#### 3.1.2 Histone methyltransferase inhibitors (HMTi)

Histone methyltransferases (HMTs) catalyze the methylation of specific histone residues, playing crucial roles in chromatin remodeling and gene expression regulation 74. These enzymes are broadly classified into SET domain-containing and non-SET domain-containing families, with the former including EZH2, G9a, and SETD8, and the latter exemplified by DOT1L 75.

EZH2, the catalytic subunit of PRC2, mediates H3K27 trimethylation, leading to gene silencing. Silencing of PPP2R2B by EZH2 has been linked to poor prognosis in HER2-positive breast cancer and HER2-targeted therapy resistance, including trastuzumab and lapatinib. Inhibitors of EZH2, including GSK126 and EPZ-6438, have been shown to restore PPP2R2B expression, thereby mitigating resistance to HER2-targeted treatments 76, 77. EPZ-6438 is currently being evaluated in a Phase 1/2 clinical trial (NCT01897571) for solid tumors and hematologic malignancies 78. G9a, a major lysine methyltransferase, catalyzes the dimethylation of H3K9 and is implicated in multiple epigenetic modifications, including H3K27 methylation 79. Inhibitors such as BIX-01294 have demonstrated the ability to promote the induction of apoptosis in breast cancer cells and reverse epithelial-mesenchymal transition, thereby reducing metastatic potential 80. Similarly, the SETD8 inhibitor UNC0379 has been reported to inhibit breast cancer cell growth by inducing DNA damage and resulting in the arrest of the cell cycle 81. DOT1L, a non-SET domain methyltransferase, regulates H3K79 methylation and is related to oncogenic gene expression. DOT1L inhibitors such as EPZ004777 have been shown to effectively suppress breast cancer growth and metastasis 82.

These HMTi not only exhibit standalone antitumor effects but also hold potential for combination therapies that enhance treatment efficacy. For example, combining EZH2 inhibitors with PARP inhibitors has been demonstrated to markedly enhance cytotoxicity in BRCA1-mutated breast cancer cells 83. Furthermore, targeting HMTs may help overcome drug resistance, offering a promising avenue for improving breast cancer treatment outcomes 84.

#### 3.1.3 Histone lysine demethylase inhibitors (HDMi)

Histone lysine demethylases are enzymes that remove methyl groups from specific histone lysine residues, thereby modulating gene expression. In accordance with their catalytic mechanisms, these enzymes are classified into lysine-specific demethylases (LSDs) and the Jumonji C (JmjC) domain-containing family 85. LSD1 was the first histone demethylase to be identified and primarily mediates the demethylation of H3K4me1/2 and H3K9me1/2 86. The JmjC domain-containing family is further divided into multiple subgroups, including the KDM5 and KDM6 families, each targeting distinct histone methylation sites 87.

Among histone demethylases, LSD1 is particularly well-studied due to its frequent overexpression in breast cancer 88. By demethylating H3K4me2, LSD1 downregulates tumor suppressor genes, while its activity on H3K9me2 promotes epithelial-mesenchymal transition (EMT), enhancing cancer cell proliferation, invasion, and metastasis 89. Inhibitors targeting LSD1, such as Tranylcypromine (TCP) and ORY-1001, have demonstrated the ability to suppress ER+ breast cancer cell growth and increase sensitivity to endocrine therapy 90, 91. The KDM5 family, including KDM5A and KDM5B, is also frequently overexpressed in breast cancer. These enzymes catalyze the removal of H3K4me3, thereby suppressing DNA damage repair and cell cycle regulation-related genes. This repression contributes to tumor progression and drug resistance 92. Notably, KDM5 inhibitors such as CPI-455 and KDOAM-25 have shown potential in reversing resistance to chemotherapy, thereby enhancing treatment efficacy 93. The role of KDM6 family members, UTX and JMJD3, in breast cancer is complex. While UTX serves as a tumor suppressor, with its inactivation leading to increased H3K27me3 levels that repress tumor suppressor genes, JMJD3 exhibits oncogenic potential by demethylating H3K27me3 and activating oncogene expression 94. The KDM6 inhibitor GSK-J4 has been found to effectively inhibit the proliferation and invasiveness of triple-negative breast cancer (TNBC) cells 95.

#### 3.1.4 Histone acetyltransferase inhibitors (HATi)

HATs play a dual role in cancer biology, functioning as both tumor suppressors and oncogenic drivers depending on the context 96. These enzymes catalyze acetyl-CoA-derived acetyl group transfer to histone lysine residues, thus altering chromatin structure and gene expression. According to their sequence homology and substrate specificity, HATs are classified into the HAT1, GCN5/PCAF, MYST, p300/CBP, and Rtt109 subfamilies 97.

HATi are broadly divided into bisubstrate inhibitors and small-molecule inhibitors 96. The clinical application of bisubstrate inhibitors is limited due to their poor metabolic stability and low cell permeability. Consequently, most currently available HATi are small-molecule compounds, many of which are derived from natural sources. For instance, anacardic acid, a naturally occurring HAT inhibitor, can markedly inhibit p300 and the p300/CBP-associated factor 98. However, the phenolic structures in many natural HATi make them susceptible to oxidation, which limits their stability. Several synthetically designed HAT inhibitors have been developed to address these limitations 99. A-485, a selective inhibitor targeting p300/CBP, has demonstrated potent anticancer activity against multiple malignancies, including colon, liver, and prostate cancers, as well as hematologic cancers 100. Another notable HATi, C646, preferentially targets p300 and has been shown to suppress the survival and invasiveness of gastric cancer cells 101. Additionally, Remodelin, an inhibitor of the acetyltransferase NAT10, has been reported to overcome doxorubicin resistance in breast cancer by reversing EMT, a key process in tumor progression 102.

### 3.2 DNA methylation inhibitors (DNMTi)

DNA methylation, a critical epigenetic modification, is primarily mediated by DNA methyltransferases (DNMTs) 103. The first epigenetic drugs developed to treat cancer were DNMT inhibitors (DNMTi), which include nucleoside analogs and non-nucleoside inhibitors 104. Nucleoside analogs function by incorporating into DNA, forming covalent complexes with DNMTs that trigger their degradation 105. Among these, Azacitidine was the first FDA-approved epigenetic therapy for cancer. This cytidine analog irreversibly binds DNMTs, leading to DNA demethylation and reactivation of silenced tumor suppressor genes 106. Another FDA-approved nucleoside analog, Decitabine, incorporates into DNA and induces hypomethylation, thereby disrupting DNA replication and promoting S-phase cell cycle arrest 107. Both Azacitidine and Decitabine have received approval as treatments for acute myeloid leukemia, chronic myelomonocytic leukemia, and myelodysplastic syndromes 108, 109. Zebularine, another cytidine analog, selectively inhibits DNMT1 and forms covalent complexes with DNMTs, effectively reversing gene silencing. Compared to other DNMTi, Zebularine exhibits greater chemical stability and lower toxicity. It has been shown to demethylate the p16 gene promoter, leading to the reactivation of the p16 tumor suppressor gene, which is frequently silenced in cancer 110. Currently, Zebularine is a potential anticancer agent for breast cancer treatment, either as a monotherapy or in combination regimens 111. Non-nucleoside DNMTi, in contrast, directly interact with the catalytic domain of DNMTs, rendering them inactive 112. However, the clinical development of this class of inhibitors remains in the early research phase. Several promising compounds, including procainamide, SGI-1027, DC-05 analogs, and derivatives of quinazoline, propiophenone, and pyrrolopyridine, are being investigated for their potential as anticancer agents 113.

## 4. Summary and Future Perspectives

Epigenetic alterations, particularly aberrant histone modifications and DNA methylation, are central to breast cancer development and therapeutic resistance. HDACi have been widely explored, but their use as monotherapies has shown only limited efficacy in clinical trials. Consequently, current research is focused on their combination together with chemotherapy and targeted treatments to enhance therapeutic outcomes. A number of clinical trials are underway.

Currently, emerging epigenetic targets and innovative drugs mainly include KAT6A/6B inhibitors, NSD2/WHSC1-targeted therapies, and super-enhancer regulation. The first-in-class KAT6 inhibitor, PF-07248144, demonstrated promising and durable anti-tumor activity in later-line treatment of ER+/HER2- advanced breast cancer. When combined with fulvestrant, the objective response rate (ORR) reached 30.2%, with a median duration of response (DOR) of 9.2 months. The median progression-free survival (PFS) was 10.7 months—significantly longer than the 1-2 months observed with fulvestrant monotherapy—and efficacy was independent of ESR1 or PIK3CA mutations 114. Histone methyltransferase NSD2/WHSC1 has been found to be highly expressed in triple-negative breast cancer (TNBC) and is associated with poor prognosis, as it accelerates tumor metastasis by promoting autophagy. Inhibition of NSD2 can block autophagic flux and significantly suppress TNBC metastasis in animal models. Small-molecule inhibitors targeting NSD2 are in the preclinical development stage 115. The targets regulated by Super-Enhancers are FOXC1 and ANLN. CRISPR/Cas9-mediated knockout of the FOXC1 enhancer or targeted inhibition of the ANLN enhancer to reduce its expression can suppress tumor growth and metastasis 116.

Despite the promise of histone modification-based therapies, a major challenge lies in their broad-spectrum activity, which can lead to off-target effects and toxicity as a result of the disruption of gene expression within healthy cells. Therefore, improving the selectivity of epigenetic drugs remains a key priority in drug development. Precision medicine demands the multi-dimensional integration of molecular data to shift from "one drug for one disease" to "one strategy for one patient." PIK3CA is one of the most prevalent mutated genes in breast cancer. For patients with hormone receptor-positive, HER2-negative locally advanced or metastatic breast cancer, PIK3CA testing should be routinely performed before first-line treatment. If mutations are detected, patients may benefit from treatment with PI3K inhibitors such as inavolisib and alpelisib 117. Future advancements in precision medicine may enable single-gene epigenetic editing approaches, allowing for highly targeted regulation of gene expression. This strategy could facilitate the development of more personalized treatments, minimizing adverse effects while maximizing therapeutic efficacy. As studies in the field of epigenetics continue to expand, the discovery of new histone-modifying enzymes and regulatory pathways will provide further opportunities for the development of targeted epigenetic therapies. These advancements hold great potential for improving cancer treatment, particularly for breast cancer, by offering novel therapeutic approaches to overcome drug resistance and enhance patient outcomes.
